# Effect of Phosphine on Coke Formation during Steam Cracking of Propane

**DOI:** 10.3390/ma14175075

**Published:** 2021-09-04

**Authors:** Manjunath Patil, Marko Djokic, Kim Verbeken, Marie-Françoise Reyniers, Kevin M. Van Geem

**Affiliations:** 1 Laboratory for Chemical Technology, Ghent University, Technologiepark 125, 9052 Ghent, Belgium; manjunath.patil@ugent.be (M.P.); Marko.Djokic@UGent.be (M.D.); MarieFrancoise.Reyniers@UGent.be (M.-F.R.); 2 Department of Materials, Textiles and Chemical Engineering, Ghent University, Technologiepark 46, 9052 Ghent, Belgium; Kim.Verbeken@UGent.be

**Keywords:** steam cracking, anti-foulants, coke formation, towards net-zero carbon emissions, heat-resistant high-temperature alloys

## Abstract

In conventional steam cracking feedstocks, contaminants such as sulfur, phosphine, and heavy metal components, present in trace levels, are believed to affect coke formation on high temperature alloys. To gain an understanding of the role of phosphine coking rates on 25/35, CrNi and Al-containing reactor materials were determined in a plug flow reactor during cracking of a propane feedstock doped with ppb levels of PH_3_ in the presence of DMDS. The presence of phosphine decreased the asymptotic coking rates by more than 20%, while it had a smaller influence on the catalytic coking rate. The coking rate was more severely reduced for the 25/35 CrNi alloy in comparison to the Al-containing alloy. The ppm levels of phosphine did not affect the olefin yields nor the production of undesired carbon monoxide. The morphology of the coked alloys were studied using an off-line Scanning Electron Microscope with Energy Dispersive X-ray detector (SEM with EDX) images of coked coupons. Two types of coke morphology are observed, i.e., filamentous coke with DMDS as an additive and globular coke in the presence of phosphine. The effect of phosphine on the material has a positive impact on the oxide scale homogeneity of 25/35 CrNi alloy, whereas the Al-containing alloy remained unchanged.

## 1. Introduction

A vast amount of research is being done to reduce coking rates during steam cracking of hydrocarbons, the dominant industrial process to produce light olefins. The top 10 chemical producing companies in 2018 had a combined turnover of €2,900 billion, which accounts for 86% of global chemical sales. These chemical sales have been growing consistently since 1988, having expanded three times in value by 2018. With increasing global sales, the necessity of reducing carbon emissions has become vital now more than ever. The long-term data indicate a decrease in total greenhouse gas (GHG) emissions by 58.4% between 1990 to 2017 [1]. The growth of this trend should further increase and emissions must fall by half by 2030 and therefore reach carbon net-zero by 2050 in order to reach the 1.5 °C goal [2]. Energy generated from a renewable source is the only contribution to the mitigation of climate change. Around 15% of energy supply from the grids in the European Union has a green label, the green energy that are produced from biomass, geothermal, wind, solar, biogas and hydroelectric sources contribute to only 3% of total world energy consumption [3]. The potential in transitioning from the use of fossil fuels as a source of energy to a “power to chemicals” approach will affect how the top commodity chemicals are produced. These chemicals form the basis of further transformation into end-products. Steam cracking furnaces are responsible for 75% of the CO_2_ emissions of a steam cracking plant. Green energy utilization and reduction in coke formation would lead to enhancements in heat transfer in the radiation section; they are the key ingredients to significantly reduce CO_2_ emissions. Steam cracking reactions in the furnaces are inevitably associated with coke deposition on the inner wall of the reactor. The steam and hydrocarbons are fed to the furnace, prior to which they are preheated in the convection section preheated by the flue gas. Depending on the feedstock, the feed is heated up to 500–680 °C to initial cracking temperatures. The gas stream enters the fired tubular reactor at this point, which are heated to temperatures of around 760–870 °C at a short residence time of 0.1–0.5 s. The cracked gas are converted into olefins, diolefins and heavier olefins. These reactions are highly endothermic in nature with huge energy demands. The olefins leaving the tubular reactor are quickly degraded by secondary reactions. In order to prevent this, the products are quenched to temperatures of around 550 °C in 0.02–0.1 s [4,5,6,7,8]. Due to the high temperature of the reactor material, the industrial standard is Fe-Ni-Cr alloys, which has high creep and heat-resistant properties. It is known that iron and nickel catalyze coke formation. The formed coke layer in the inner wall of the reactor acts as an insulation to heat transfer and eventually increases the thickness of the coke, causing a high pressure drop and leading to the shut-down of the furnace for decoking the carbonaceous deposits on the wall of the reactor.

To sustain more severe operating conditions, the furnace coil suppliers have done an enormous amount of work in developing new techniques for producing reactors and finding new material for the construction of steam cracking reactions [9,10,11,12,13,14,15,16,17,18].

The severe operating conditions employed in steam cracking furnaces enable the production of higher yields of valuable products. Modified coils can help achieve these conditions, but the design of coils are limited by metallurgy. A wide variety of iron- and nickel-based materials are normally used in the construction of fittings, piping, valves, pressure vessels and other equipment in refineries and petrochemical plants, among which the most common is carbon steel. Carbon steel can only operate at temperatures below 350 °C, as it is susceptible to oxidation and therefore loss of strength. Up to 9% of chromium and up to 1% of molybdenum can be added to improve their operating temperatures to up to 650 °C. Specialized Ni-Cr-Fe alloys are required at higher temperatures in order to improve their corrosion or carburization resistance. The cast- and heat-resistant alloys with a high Cr and Ni content are normally used in these aggressive environments of petrochemical and refining applications. These wrought and heat-resistant alloys were used solely used in the reformer tubes for 20 to 25 years, and were replaced by an earlier HK40 material. Gradually, the HK40 was replaced by HP-Nb and most recently, 35Cr-45Ni has been employed [19]. A recent generation of alloys has been enriched with aluminum to improve coking rates and enhance resistance to carburization. The attention to the limitation in the addition of Al to the alloys is required to avoid low-melting point compounds and to decrease the creep resistance properties of the alloys. Comprehensive research by Munoz et al. [20] is available, where they tested nine commercially available materials for ethane steam cracking under industrially relevant operating conditions. The Al-enhanced alloys and NiCr alloys were compared with silicon carbide. The overall results indicate that the Al-enhanced alloys are better in resistance to coke formation compared to NiCr alloys. The coking rates were reduced in comparison to those with a layer of manganese chromate on the surface of the alloy [20]. Asteman et al. [21], on the other hand, indicated that too high an aluminum content in the alloys is not so beneficial. They tested the behavior of commercial spun-cast Ni-based alloys and Al_2_O_3_ former on the impact of the Al and Cr content on the oxidation behavior. The results showed that after 500 h of isothermal exposure, the alumina former containing 4 wt % Al had the best oxidation properties, with a minimum spallation of oxide layers compared to chromia-forming alloys. On the contrary, the NiCr28 showed substantial oxidation and Cr-evaporation. The authors tried to improve the oxidation properties by adding a greater Al content to the alloys (NiCr25Al10); the alloy failed to form uniform Al_2_O_3_ oxide. The oxide layer had individual ‘‘grains’’ of the two matrix phases from different mixed non-protective oxides, one consisting of a Cr/Ni/Al oxide and the other of a Cr/Ni/Fe oxide.

A simple operational method to alter the environment inside the reactor in order to reduce the coking rates, is used to add specific anti-fouling compounds to the feed. Promising research has been done regarding the addition of organophosphorous compounds. Results include a noticeable decrease in the coking rate and different coke morphology and compositions within the coke layer is obtained.

The method of adding antifoulants in cracking furnaces involves the treatment of feedstock with at least 25–100 ppm of the phosphorus compounds and also to help clean up previously fouled surfaces [22]. Several different organophosphorous compounds have been studied regarding this topic. The influence of triphenyl phosphine (TPP), tri-o-tolyl phosphine (TTP), and triphenyl phosphine oxide (TPPO) on the coking rate during steam cracking of naphtha has been studied by Niaei et al. in 2008 [23]. The conclusions were that all compounds inhibited the coking rate, proportional to the amount of organophosphorous compounds added. When more than 300 ppm of the latter compounds are added, the coking rate remains constant, at a value about 5 times lower compared to when no additional compounds are added. Addition of phosphorus compounds also significantly increase runlengths, throughput and severity, which was observed by the COKE-LESS^®^ program reported by Nalco/Exxon Energy Chemicals, L.P. The inhibitor also reduced significantly the CO and CO_2_ yields during the start-up and throughout the run. The program was tested both on liquid and gas feedstocks and showed promising results [24].

Similar results were obtained by Ghosh and Kunzru in 1988 [25] for the compound triethylphosphite (TEP) and by Kunzru and Chowdhury in 1993 [26] for benzyl diethyl phosphite (BDP). The coke formation reduction increased initially, until at higher concentrations, the coking rate approached a constant value. The same conclusion was made, i.e., the phosphorous-containing additives passivate the alloy surface. When 200 ppm of BDP is added to the naphtha feed, the asymptotic coking rate is reduced by 87.7%. The study, however, lacked evaluations under additive concentrations less than 1 ppm wt % or even 50 wt % with differences in alloy types.

Another beneficial effect of phosphorous addition is the altering of the morphology and metal content of the cokes being formed. Cokes formed in the presence of phosphorous are typically finer and more porous compared to the reference case without inhibition. This is industrially preferred since the cokes can be removed more easily during decoking, reducing the decoking time. Lastly, it is proven by Niaei et al. [23] that the amount of metal particles in the cokes decreases when phosphorous is used as an inhibitor. According to the literature, the gas phase hydrocarbons first chemisorb on the metallic surface of the reactor, thereby losing hydrogen atoms which recombine and desorb into the gas phase. Surface carbon atoms are formed and can dissolve through a metal particle by precipitation. Due to precipitates on the active sites, the carbon exerts pressure on the metal particles. When this pressure becomes stronger than the tensile strength of the metal, the carbon is able to lift a metal particle from the surface. Carbon stems arise and the carbon whisker or filament keeps growing, pushing the catalytic particle further away from the surface [27,28,29]. However, the research lacked quantitative information regarding the catalytic and asymptotic coking rates of aged alloys and the comparisons of the behavior of reactor tube alloys.

Propane is chosen as a hydrocarbon feedstock because it is a widely used feedstock and it is a product from natural gas production, oil production and shale gas production. Whatever the source of the natural gas, once separated from the other hydrocarbons in a depropanizer combined with a C3 splitter, it is commonly sent to a steam cracker or a propane dehydrogenation unit. Steam crackers can operate with not so pure propane and some contaminants can be present. The raw natural gas contains water vapor, hydrogen sulfide, carbon dioxide, nitrogen and other ultra-trace (ppb levels) components such as phosphine, arsine, mercury, etc. [30] These trace contaminants have a direct effect on the coking in the reactor coils during steam cracking. Due to the boing point of phosphine impurities, they always end up in propane after a C3 splitter. The purpose of this work is to mimic the industrial conditions for coke formation with contaminants such as sulfur and phosphorus compounds that can be present in propane at ppm and ppb levels. In particular, we have evaluated their impact on coking rates during steam cracking. These experiments are carried out in a thermogravimetric unit, consisting of a plug flow reactor (PFR), which allows us to evaluate coke formation in real time behavior on a 25/35 NiCr and Al-containing reactor material with phosphine as a contaminant. The coking rates are evaluated under industrially relevant tube metal temperatures similar to start-of-run and the end-of-run tube outlet cracking conditions in a plug flow reactor set-up. The coked alloys/coupons after steam cracking are studied off-line using Scanning Electron Microscopy with Energy Dispersive X-ray microscopy (SEM–EDX) to understand the effect of contaminants exploiting as antifoulants on a protective barrier scale and the microstructure in the bulk matrix. The results shine new light on the impact of phosphorous-containing additives.

## 2. Experimental Section

**Figure 1:**
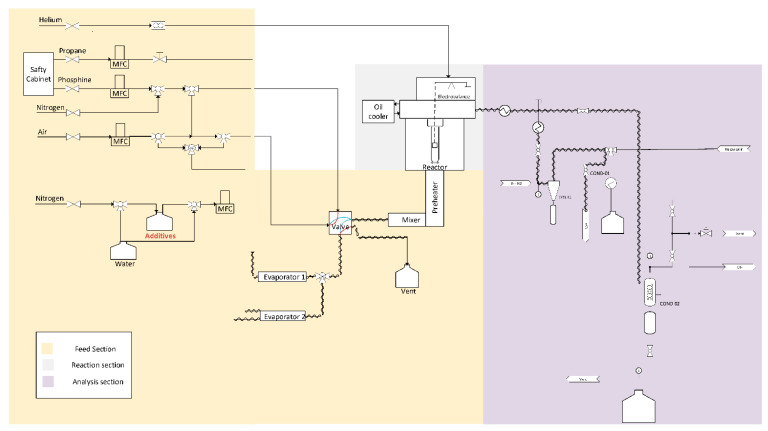
Schematic of plug flow reactor unit equipped with an electro-balance for the study of coke deposition on different reactor materials.

A plug flow reactor setup is used to study coke formation that consists of three parts: the feed section (with preheaters), reaction section and analysis section (see Figure 1). All feed streams are controlled with mass flow controllers to achieve the desired flow rates. The plug flow reactor is made out of quartz. Within it, a metal coupon hangs from the arm of an thermogravimetric balance. The latter measures the mass changes over time due to the coke deposition on this coupon and the used frequency is 1 Hz. An Refinery Gas Analyzer (RGA, Agilent – 6890N, Belgium) with a thermal conductivity detector (TCD) for detection of permanent gases (N_2_, H_2_, O_2_, CO and CH_4_) and C2-components and a flame ionization detector (FID) for detection of hydrocarbons was used. This GC is used to analyze the C_4_ minus components of the gas stream. To inject effluent into the RGA, a condenser that uses propylene glycol at 278 K as cooling media is present, which ensures only the permanent gases reach the chromatography. A Ultra GC (Thermo Fisher Scientific – TRACE^TM^, Belgium) equipped with an FID detector was also used to analyze C1 to C10 hydrocarbons.

The range for the elemental composition of the materials used as coupons are shown in Table 1. Since it is impossible to make a reactor out of each alloy to be investigated, a coupon of the respective alloys are fabricated and suspended in the reactor.

The coupon dimensions are 8 mm × 10 mm × 1 mm, cut from the inner side of an industrial coil using a wire-cut Electrical Discharge Machining (EDM) (University of Gent, Zwijnaarde, Belgium) with an accuracy of 1 μ, is suspended from the arm of an electro-balance. The Kanthal wire is connected to the arm of a Cahn D-200 electrobalance (Thermo Electron, Brussels, Belgium) and the coupon, which continuously measures the mass of the coupon as a function of time. During the experiments, the balance is protected by a small overpressure in the chamber by helium gas.

The coke deposition on each coupon is measured over time. This allows the determination of the absolute amount of coke deposited during every cracking cycle, as well as the calculation of the initial and the asymptotic coking rate.

**Figure 2:**
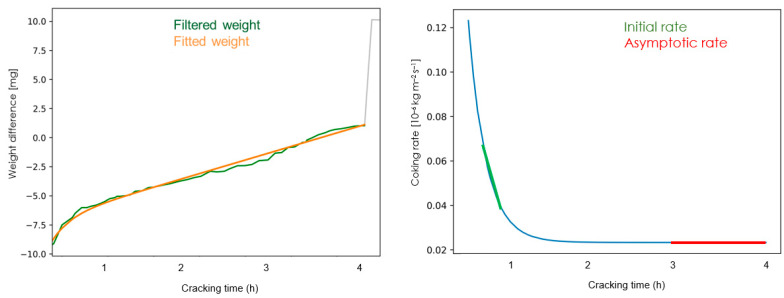
The filtered and fitted signal (**left**) and the derivative of the mass gain curve representing the coking curve rate (**right**).

The experimental data are logged during the experiment and stored in an Excel file. The raw data are filtered and fitted with a moving average and the derivative of the mass gain is executed on a Python program (Version 3.7, University of Gent, Zwijnaarde, Belgium) as shown in Figure 2. The filtered weight curve is then regressed by minimizing the total sum of squares to the following Equation wither parameters *A*, *B*, *C* and *D*:

(1) $$W=At+B\left(1-\frac{1}{2}\left(e^{-Ct}+e^{-Dt}\right)\right)$$

This represents the fitted weight curve, with W the amount of coke [10^−3^ g] [31] on the surface at time t. The data points for the first 15 min are calculated via the fitted weight curve. The corresponding coking rate curve can be obtained by differentiating the equation above:

(2) $$r_{c,coupon}=\frac{dW}{dt}=A+\frac{B}{2}\left(Ce^{-Ct}+De^{-Dt}\right)\;\;\;\;\;\left[\frac{10^{-3}g}{h}\right]$$

The reported coking rate is then obtained by converting to the desired units:

(3) $$r_{c}=\frac{1000}{3600\cdot S_{coupon}}\cdot r_{c,coupon}\;\;\;\;\;\left[\frac{10^{-3}g}{s\cdot m^{2}}\right]$$

With *S_coupon_* the surface area of the coupon. The coking rate can also be calculated as a discrete derivative from the fitted weight curve:

(4) $$r_{c}=\frac{1000}{3600\cdot S_{coupon}}\cdot \left(\frac{W_{t_{i}}-W_{t_{i-1}}}{t_{i}-t_{i-1}}\right)\;\;\;\;\;\left[\frac{10^{-3}g}{s\cdot m^{2}}\right]$$

The initial coking rate reported is the mean measured value between the initial 15 and 60 min and is used to characterize the catalytic activity of the sample. The initial coking rate is mainly representative of the metal-catalyzed reactions [32]. The asymptotic coking rate is related to the radical coking mechanism and is reported as the mean measured coking rate between the 3rd and 4th hour of cracking.

### Procedure and Chemicals

The experimental work is divided into two sections: with phosphine as additive and without phosphine as an additive (reference case) during steam cracking of propane. The experiments are performed using two different alloys; 25/35 CrNi and Al-containing alloy. For both alloys, the coupons are cut from the inner side of an industrial coil using wire-cut Electrical Discharge Machining (EDM) with an accuracy of 1 µm. The operating conditions for the experiments with and without phosphine are summarized in Table 2 and Table 3. Both the cyclic aging of the materials in the process steps are very similar, the only difference being the addition of phosphine from 188 vol-ppb (or 228 ppb PH_3_ m/m) phosphine diluted in nitrogen gas cylinder supplied by Air Liquide^®^ (Air Liquide, Antwerp, Belgium). The total mass flow to the reactor, i.e., propane, steam and nitrogen (with or without phosphine gas mixture), remains the same for both the experiments. In case of a reference experiment: only nitrogen is fed to the reactor compared to nitrogen and phosphine mixture for the latter experiment. Before the first cracking cycle, pretreatment with steam is performed, oxidizing the alloy surface: The temperature of the reactor is increased from 573 K to 1223 K for 13 h in a steam environment. This is followed by a presulfiding step (DMDS conc. of 500 ppmw/H_2_O) at 1100 K for thirty minutes. All the experiments consist of two coking cycles. The first 4 h of steam cracking is performed at a gas-phase temperature of 1223 K. This is followed by increasing the temperature of the reactor at 375 K/h to 1323 K for a duration of 45 min. This is to mimic the metal temperatures corresponding to start-of-run and end-of-run industrial conditions, respectively, consequently aging the material. As the next steps after the first steam cracking cycle, the pretreatment procedure consists of a steam/air treatment. This is performed in order to burn the coke formed on the sample and regenerate the oxides formed on the sample surface. After steam cracking for four hours and forty-five minutes, the reactor temperature is set to 1073 K and the flow rate of ethane and steam is set to zero, leaving only helium to purge through the reactor as a standby. After the second coking cycle, for the reactor cooling down to room temperature, an He flow of 0.6·10^−6^ kg·s^−1^ is used as an inert gas, while the furnace ramp is set to 50 K per hour.

## 3. Results and Discussion

### 3.1. Coking Rates

The overall experimental conditions and the coking rates for 25/35 CrNi and Al-containing alloys are shown in Table 4. The results show that the effect of phosphine is more prominent on the 25/35 CrNi alloy compared to the Al-containing alloy.

Firstly, with the catalytic coking rates, the classical 25/35 CrNi alloy shows a decrease in coking rate in the first coking cycle by 14% in the presence of phosphine in contrast to the reference experiment (without phosphine addition). The second coking cycle behaves similarly, a decrease in 14% of catalytic activity is observed. The Al-containing alloy shows a decrease in catalytic coking rates by 12% in the presence of phosphine in the first coking cycle and 10% in second coking cycle compared to the referenced experiments.

The 25/35 CrNi alloy, when compared to the reference experiments, shows decreased catalytic coking rates in the presence of phosphine by 25% and 33% in the first and second coking cycle, respectively. The increase in asymptotic coking rate observed between the first and second coking cycle decreases from 18% to 8% in the presence of phosphine. Similar observations were made for the Al-containing alloy: the coking rates are lower by 16% and 24% in the first and second coking cycles when compared with the reference experiment. This represents a decrease in asymptotic coking rate by 15% in the presence of phosphine between the first and second coking cycle and only a 6% decrease in the case of the reference experiment. There was no influence on the product yields and especially observed was that the carbon oxides were stable in both cases, i.e., with and without phosphine in the propane feed. The initial low concentration of phosphorous precursors chemisorbed on the coupon during the first forty-five minutes under steam cracking conditions explains the lower effect on initial coking rates compared to its effect on the asymptotic coking rates between the third and fourth hour of steam cracking conditions.

The performance of three different Cr/Ni alloys along with Al-cont. samples were evaluated using a jet-stirred reactor [33]. It was observed that the worst performance is from the 25/35 CrNi alloy. The Al-cont. alloy had two to three times more coke than the other non-aluminum containing alloys tested in the absence of DMDS. Therefore, dosing the optimal amount of DMDS is crucial for all the tested alloys. At initial pre-oxidation temperatures of 1023 K, the best performance was observed for the CrNi alloy with the highest Cr content and the worst performance shown for the 25/35 CrNi alloy, with a continuous addition of DMDS. The alloys tested with continuous addition runs had a positive effect on coking rates, with the application of presulfiding compared to Blank runs. Cyclic aging of the alloys in the presence of DMDS had an effect on the CrNi alloys, high chromium and manganese content and oxide spallation for the 25/35 CrNi and 35/45 CrNi alloys were observed. The coking rates observed with the plug flow reactor, shown in this work, has increased catalytic coking rates for the 2nd cracking cycle by 25% compared to the coking rates observed with the jet-stirred reactor. This can be attributed to the high coupon temperature. The effect of pretreatment on Al-containing alloys is a significant impact on the coking rates, which were observed to improve by a factor of 5. Metallurgical aging evaluation was performed by Sarris et. al. [34] The coking behavior of four different alloys after evaluating in a thermogravimetric set-up after their exposure to a long-term high temperature oxidation for 500 h was observed. The results indicated that, for the same conditions, the coking resistance is improved by increasing the content of Cr and Ni of a high temperature alloy. Mainly, the 25/35 Cr/Ni alloy showed pronounced coking rates after cyclic aging. However, it should be noted that most studies previously performed have been with ethane and not propane. Due to its higher propylene content, the structure of coke is different.

### 3.2. SEM and EDX Analyses

The coked samples were analyzed using Scanning Electron Microscopy (SEM), with an Energy Dispersive X-ray (EDX) using a JOEL analyzer, type JSM 7600F (University of Gent, Zwijnaarde, Belgium). The analysis is performed to study the cross-sectional microstructure of the oxide layers and the coke morphology at the surface of the sample. All the samples were initially analyzed at the surface, followed by imbedding the samples using a Polyfast^®^ resin (University of Gent, Zwijnaarde, Belgium). The samples are then etched electrolytically with 6V for 10 s using 10% Nital (10% vol. nitric acid in ethanol from University of Gent, Zwijnaarde, Belgium) for a cross-sectional study.

**Figure 3:**
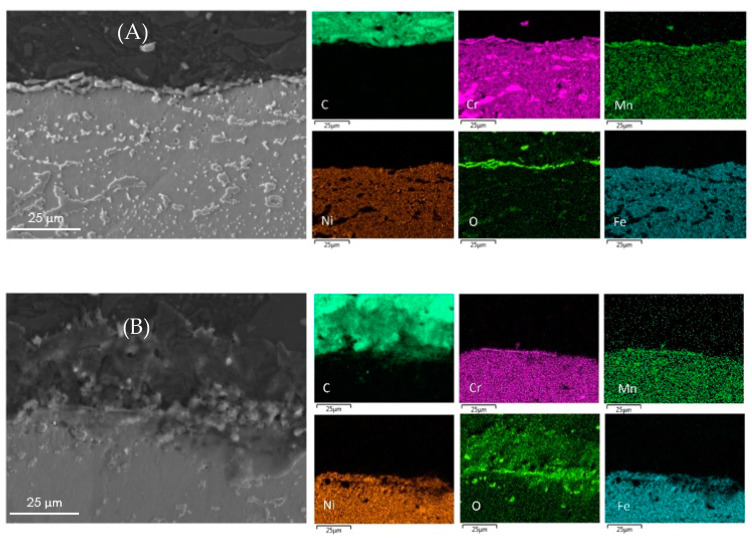
Cross-sectional mapping with SEM and EDX on coked 25/35 CrNi alloys with (**A**) DMDS and phosphine as additives; and (**B**) only DMDS as an additive.

The surface elemental composition of all coked samples are summarized in Table 5. The metal content obtained by EDX pertains to a small surface fraction only. The metal content and composition of the coke layer can vary from point to point and the values presented are the mean values of five analyses at various locations over the surface. The cross-sectional mapping of the coked 25/35 CrNi alloys in the presence of phosphine and DMDS is shown in Figure 3. From Figure 3A, it can be seen that the chromium layer at the surface is continuous with corresponding manganese and oxygen, which is sharply defined with a thickness of approximately 1µm. The elemental surface composition using EDX of the coked 25/35 CrNi alloy with the continuous addition (CA) of DMDS and phosphine, from Table 5, shows a high concentration of chromium and less than 19 wt % of Fe and Ni together. These lower amounts of catalytically active species, Fe and Ni, inhibit the overall coking rates over the run length and due to their low concentrations, further growth of filamentous coke is reduced.

In the case of a 25/35 CrNi alloy with only DMDS as an additive, from Figure 3B and Table 5, chromium and manganese oxide layers at the surface of the sample appears to be irregular, leaving the bulk matrix exposed to a gas stream. The overall concentration of chromium at the surface is 53% lower, compared to the sample exposed to the continuous addition of phosphine together with DMDS, whereas the amount of Fe and Ni particles are increased by a factor 3.

**Figure 4:**
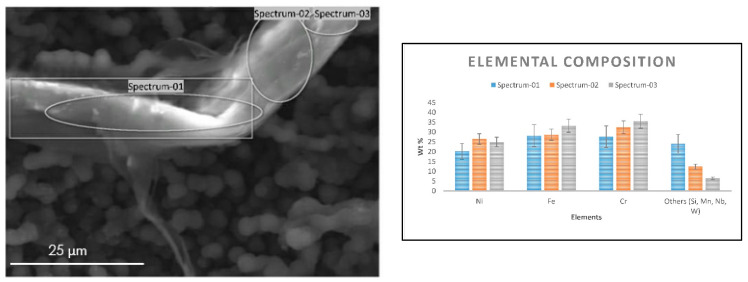
SEM image of the coke filament from the 25/35 CrNi alloy (**left**) and its elemental composition (**right**) recorded with an accelerating voltage of 10 kV after two 5 h coking cycles with DMDS as an additive.

Similar observations were noted on a coke filament from 25/35 CrNi alloy, which was analyzed using an EDX detector, as shown in Figure 4. Most of the carbon filaments were observed with a diameter between 3–6 µm and length of 40–60 µm. The coke filament in Figure 4 appears to be intertwined between filaments on the surface that has been in contact with the gas stream with a diameter of about 15 µm. The presence of chromium on the coke filament is due to the particles of an oxide scale that spalled during steam cracking and grew along with the coke layer. However, a local elemental composition of Ni and Fe particles in Figure 4 appears partially encapsulated by coke deposition, which is in line with the global composition of the sample measured over the surface in Table 5 despite the abnormalities of the coke structure seen on SEM. Since the analyses are performed on the coke samples, it has to be mentioned that for the same accelerating voltage the penetration depths can vary, as the thickness of the coked sample are not equal. The influence due the material properties is illustrated by Kanaya-Okayama [35]. The calculated nominal thickness of the coke with only DMDS as an additive for 25/35 CrNi and Al-cont. coupons are 28.4 µm and 4.3 µm, respectively. The penetration depths of accelerating voltage at 10 kV and 20 kV for 25/35 CrNi and Al-cont. are 1.52 µm and 1.61 µm using 10kV and 5.31 µm and 5.48 µm respectively. Therefore, the accelerating voltage of 10kV was chosen as a better representation of the surface and is used for a comparison of the results.

**Figure 5:**
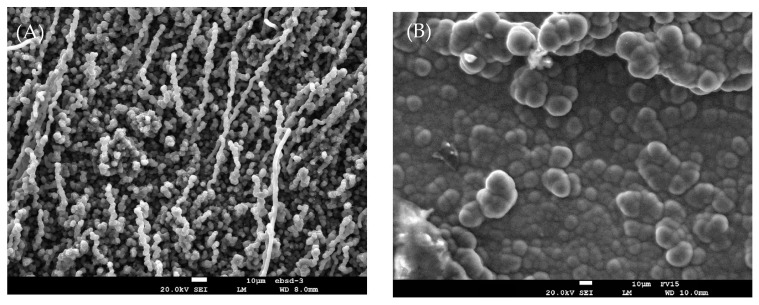
The surface of two coked 25/35 CrNi alloys after application of (**A**) DMDS as an additive; and (**B**) DMDS and phosphine as an additive and two 6 h coking cycles with an accelerating voltage of 20 kV.

**Figure 6:**
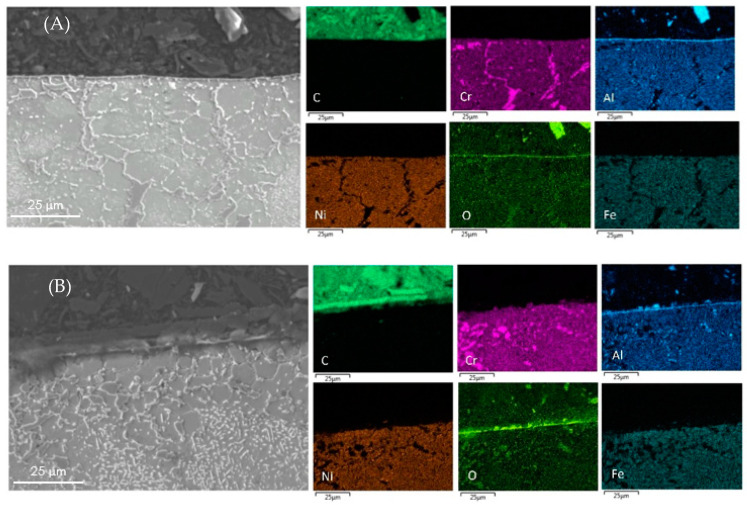
Cross-sectional mapping with SEM and EDX on coked Al-containing alloys with (**A**) DMDS and phosphine as additives; and (**B**) only DMDS as an additive.

SEM photomicrographs of the coke deposited on a 25/35 CrNi alloy after application of DMDS and DMDS with phosphine as additives is shown in Figure 5. Two types of coke morphology can be observed, i.e., coke filaments in Figure 5A and coke globules in Figure 5B. In Figure 5B, the surface in contact with the gas-stream is covered by globular coke and the presence of some metal particles can be visualized. It can be seen that the metal particles are found either within the filamentous carbon (Figure 4 and Figure 5A) or on the top of the coke structure, and the population of globular coke is much lower than carbon filaments. The results are in line with the research reported from Baker, Marek and Pradip Das [36,37,38]. Pradip Das studied the effect of concentration of benzyl diethyl phosphite on coking rates. These authors reported that low additive concentrations in the feed is more appreciable, and increasing concentrations of phosphorous additives resulted in a change in coke morphology from globular to a more filamentous structure. With an increasing time on stream or deactivation of active species due to added additives, the metal surface is encapsulated by coke and the catalytic activity of the metal wall decreases. The main route for coke formation then shifts to the heterogeneous non-catalytic mechanism, where the coke is formed through reactions of coke precursors in the gas phase and the active sites are located in the coke matrix [39,40,41]. In Figure 6A,B, a clear homogeneous alumina layer of approximately 0.5 *µ*m thickness is formed at the surface of the sample, in both cases, in contrast to the chromia layers formed in 25/35 CrNi alloys. The same alloys were used in the evaluation of phosphine, as previously performed research shows [42]. To evaluate the crystal structure of the coupons, XRD analysis was performed. The crystalline phases were determined using a Siemens Diffractometer Kristalloflex D5000, with Cu Kα (λ = 0.154nm) radiation. The XRD patterns were collected in a 2θ range from 10° to 80°, with a step of 0.04°. Since the patterns of the pretreated samples show mainly three big peaks (Fe-Ni to an angle of 43.65°, 50.8° and 74.75°), the main patterns were Fe-N and the shoulder next to them is Ni-Cr-Fe. It is also noted that MnFe_2_O_4_ and Cr_2_O_3_ are increased by increasing the pretreatment duration and on which the current work is based. The observed Fe and Ni species, in comparison to different pretreatment techniques, were lower and were in line with the observed EDX analysis.

From Table 5, EDX measurements on Al-containing alloys shows the decreased amounts of Fe and Ni particles by a factor 3 at the surface of the coked samples compared to 25/35 CrNi alloys. However, the presence of phosphine does not, or only marginally, has an effect on the amount of active species for the Al-containing alloy. The authors were unable to replicate SEM images of an Al-containing alloy similar to the 25/35 CrNi alloy due to spallation of coke while dismantling the coupon from the setup. Aluminum oxide is stable at higher temperatures and at the performed pretreatment conditions before the coking cycles; the developed alpha-alumina layer at the surface reduces the exposure of the bulk matrix from the gas-phase stream, thereby reducing the catalytic activity. Although phosphine has clearly a positive effect on coking rates, its effect is less pronounced for the Al-containing alloy compared to the 25/35 CrNi alloy.

In the case of the Al-containing alloy, samples pre-oxidized at 1223 K in air for 12 h showed that the amount of Fe and Ni on the surface is reduced by 60% as compared to the work from Munoz et al. [20] After aging the alloy in reducing the hydrocarbon environment, according to their results, a thin, but uniform, α-Al2O3 layer is formed on top of the surface, which is stable and has no observed oxide spallation. Consequently, the unexposed bulk matrix covered by an alpha alumina layer from the surrounding hydrocarbon gas stream makes the Al-containing alloy better in terms of coking rates than the rest of the alloys. The protective chromium oxide layer becomes unstable and transforms to chromium carbide. Therefore, it causing the increased coking rates. It is observed from previous research [43,44,45] through thermodynamic calculations performed with Ekvicalc^®^ that MnCr_2_O_4_ and Cr_2_O_3_ act as a barrier against coke formation. However, according to the literature [46,47], it should be noted that in the case of NiCr alloys, chromium oxides are converted to carbides and MnCr_2_O_4_ are reduced to MnS and MnO at temperatures above 930 °C. The overall combination of aluminum addition to the alloy and continuous addition of phosphine and DMDS during steam cracking of propane results in lower and stable coking rates.

## 4. Conclusions

The influence of phosphine addition on coking tendency of 25/35 NiCr and Al-containing alloys is assessed in a plug flow reactor equipped with an electro-balance, under industrially relevant propane steam cracking conditions. Surprisingly, even small additions of phosphine reduced the measured coking rates for both alloys. The lower coking rates were observed because the phosphine is believed to react with steam to yield an oxide of phosphorous, e.g., P_2_O_5_ or elemental phosphor, which is vaporous at cracking temperatures. These vapors or elemental phosphor chemisorbed on the walls of the furnace tubes take up the active sites, and therefore contributed to lower coking rates. Relative to a reference experiment, the change in coking rates in the presence of phosphine and DMDS was:

(1) For the 25/35 NiCr: decrease in catalytic coking rate by 14% in the first and second coking cycles, asymptotic coking rates reduced by 25% and 33%, respectively.
(2) For the Al-containing alloy: the catalytic coking rates were lower by 12% and 10% in the first and second coking cycles and asymptotic coking rates were reduced by 16% and 24%, respectively.

Offline analyses with SEM and EDX mapping show two types of coke morphologies, i.e., coke filaments (DMDS as additive) and coke globules (phosphine as additive). With only continuous addition of DMDS, coke filaments have a diameter between 3–6 µm and a length of 40–60 µm and metal particles are observed within the filament. The cross-sectional analysis of 25/35 CrNi showed a homogenous oxide layer at the surface in the presence of phosphine. The Al-containing alloy had no change on the oxide scale with or without phosphine addition. The elemental composition on the surface of the 25/35 CrNi alloy with DMDS and phosphine shows a high concentration of chromium and less than 19 wt % of Fe and Ni together. These lower amounts of catalytically active species, Fe and Ni, inhibit the overall coking rates over the run length. Due to their reduced concentrations, the growth of filamentous coke slows down with CA of phosphine. Therefore, the rate of coke formation in the presence of phosphine and DMDS helps in reducing the coke formation and CO_x_ yields.

## Figures and Tables

**Table 1:** The elemental compositions of used coupons in this work.

Materials	C	Mn	Si	P	S	Ni	Cr	Al	Additions
25/35 CrNi	0.35/0.60	1.00/1.50	1.00/2.00	-	-	33/38	23/28	-	Nb, Ti, and others
Al-cont.	0.6	-	1	0.03	0.03	29/46	22/28	2/4	Nb, Ti, and others

**Table 2:** Detailed experimental conditions without phosphine as a reference scenario for CrNi and Al-containing alloys.

*Process Steps*	*Duration* *(h)*	*Gas-Phase* *Temperature* *(K)*	*Gas Feed Flow (10^−6^ kg s^−1^)*			*Steam Flow* *(10^−6^ kg s^−1^)*
			Propane	Air	Nitrogen	
*Pretreatment*	13.00	1223	-	-	-	8.33
*Presulfiding*	0.50	1100	-	-	-	
*1st CC*	4.00	1223	20.83	-	6.66	
	0.75	1323	20.83	-	6.66	
*Decoking*	0.50	1073	-	8.33	-	
*Presulfiding*	0.50	1100	-	-	-	
*2nd CC*	4.00	1223	20.83	-	6.66	
	0.75	1323	20.83	-	6.66	

**Table 3:** Detailed experimental conditions in the presence of phosphine for CrNi and Al-containing alloys.

*Process Steps*	*Duration* *(h)*	*Gas-Phase Temperature* *(K)*	*Gas Feed Flow (10^−6^ kg s^−1^)*				*Steam Flow* *(10^−6^ kg s^−1^)*	
			Propane	Air	N_2 + PH3_	Phosphine		
*Pretreatment*	13.00	1223	-	-	-	-	8.33	
*Presulfiding*	0.50	1100	-	-	-	-		
*1st CC*	4.00	1223	20.83	-	6.66	1.38 × 10^−6^		
	0.75	1323	20.83	-	6.66	1.38 × 10^−6^		
*Decoking*	0.50	1073	-	8.33	-	-		
*Presulfiding*	0.50	1100	-	-	-	-		
*2nd CC*	4.00	1223	20.83	-	6.66	1.38 × 10^−6^		
	0.75	1323	20.83	-	6.66	1.38 × 10^−6^		

**Table 4:** Summary of the experimental conditions, coking rates and product yields.

Experiment		DMDS with PH_3_		Only DMDS	
		Al-Content	25/35 CrNi	Al-Content	25/35 CrNi
Conditions	Propane flow (10^−6^ kg s^−1^)	20.83		20.83	
	Steam flow (10^−6^ kg s^−1^)	8.33		8.33	
	CA DMDS (ppmw S/HC)	41		41	
	CA PH_3_ (ppbw P/HC)	66		-	
	Temperature (K)	1223		1223	
	Pressure (barg)	1.02		1.02	
	Dilution (kg _H2O_/kg _C3H8_)	0.4		0.4	
Catalytic coking rates (10^−6^ kg m^−2^s^−1^)	1st Coking cycle	2.38	3.01	2.71	3.51
	2nd Coking cycle	2.38	3.54	2.65	4.12
Pyrolytic coking rates (10^−6^ kg m^−2^s^−1^)	1st Coking cycle	1.38	1.45	1.65	1.95
	2nd Coking cycle	1.17	1.57	1.54	2.35
Component yields (wt %)	-	DMDS with PH_3_		Only DMDS	
	-				
H_2_	-	1.93		1.90	
CO_2_	-	0.01		0.01	
CO	-	0.01		0.02	
CH4	-	25.02		24.74	
C2H6	-	1.79		1.78	
C2H4	-	37.71		37.48	
C3H8	-	11.32		11.93	
C3H6	-	7.99		8.07	
C2H2	-	2.88		2.84	
1,3-C4H6	-	2.41		2.38	
Benzene	-	4.27		4.22	

**Table 5:** Top surface elemental composition of the coked samples with EDX detector at an accelerating voltage of 10 kV.

Elements (±2 wt %)	DMDS with Phosphine		Only DMDS	
	25/35 CrNi	Al-Cont.	25/35 CrNi	Al-Cont.
Ni	7.3	4.5	23.8	4.6
Fe	10.9	7.2	26.3	7.9
Cr	78.6	14.8	36.6	15.1
Si	2.3	4.1	8.6	4.3
Mn	0.89	1.2	2.5	2.89
Nb	0.01	-	2.2	-
Al	-	68.2	-	65.21

## Nomenclature

A	Atomic mass of element [g.mol^−1^]c
DMDS	dimethyl disulfide
EDX	Energy Dispersive X-ray analysis
FID	Flame Ionization Detector
F	Flow rate [10^−6^ kg s^−1^]
H	Penetration depth [µm]
CC_i_	Coking-decoking Cycle number, i
PFR	Plug flow reactor
L	Nominal thickness [μm]
m_tj_	mass of coke at time j [kg]
MOT	maximum operating temperature [K]
Mc	amount of coke deposited on the sample [g]
P_tot_	reactor pressure [MPa]
r_c_	coking rate [kg·s^−1^·m^−2^]
r_c,initial_	initial catalytic coking rate [kg·s^−1^·m^−2^]
r_c,asymptotic_	asymptotic coking rate [kg·s^−1^·m^−2^]
RGA	Refinery Gas Analyzer
S	surface area of the coupon [m^2^]
SEM	Scanning Electron Microscopy
TCD	Thermal Conductivity Detector
T	temperature [K]
V	Accelerating voltage [kV]
z	Atomic number
